# A Virtual Living Lab Platform Codeveloped for Mental Health in Youth-Onset Type 2 Diabetes (BrightSpark Care Lab): Protocol for a Mixed Methods Study

**DOI:** 10.2196/83865

**Published:** 2026-01-19

**Authors:** Mandy M. Archibald, Brandy Wicklow, Elizabeth Sellers, Arlene Griffiths, Linda Diffey, Jonathan McGavock, Leslie E. Roos, Alexander M. Clark, Jennifer Lopez, Josephine Ho, Ernestine Ledoux, Gifty Dzorka, Shahina Parvin, Oluwatoyosi Fagbuyi, Allison Dart

**Affiliations:** 1 College of Nursing Rady Faculty of Health Sciences University of Manitoba Winnipeg, MB Canada; 2 Diabetes Research Envisioned and Accomplished in Manitoba Children's Hospital Research Institute of Manitoba Winnipeg, MB Canada; 3 Department of Pediatrics and Child Health, Max Rady College of Medicine Rady Faculty of Health Sciences University of Manitoba Winnipeg, MB Canada; 4 Community Representative Winnipeg, MB Canada; 5 Rady Faculty of Health Sciences College of Community and Global Health University of Manitoba Winnipeg, MB Canada; 6 Partnering for Research Innovations in Mental Health Children's Hospital Research Institute of Manitoba Winnipeg, MB Canada; 7 Department of Psychology Faculty of Arts University of Manitoba Winnipeg, MB Canada; 8 Office of the President University of Athabasca Athabasca, AB Canada; 9 Youth Co-researcher Winnipeg, MB Canada; 10 Department of Paediatrics Cummings School of Medicine University of Calgary Calgary, AB Canada; 11 Diabetes Clinic Alberta Children's Hospital Research Institute Calgary, AB Canada

**Keywords:** type 2 diabetes, mental health, youth, patient-centered research, living labs, arts-based knowledge translation

## Abstract

**Background:**

Type 2 diabetes (T2D) is a complex chronic disease that poses significant mental health challenges to affected youth. Despite calls for youth-centered research in this area, qualitative and mixed methods research is lacking, and longitudinal understandings of the mental health experiences of youth have not been generated. Living labs have potential as interactive knowledge exchange and longitudinal research platforms to generate such understandings.

**Objective:**

The proposed research aims to (1) codesign, with youth and parent coresearchers, a virtual living lab platform with an embedded registry of youth with T2D; (2) use this platform to generate longitudinal understandings of youths’ mental health experiences; (3) identify youth priorities for research and care based on the thematic data; and (4) codesign an arts-based knowledge translation (KT) resource to communicate these priorities.

**Methods:**

This study proposes a three-stage longitudinal, qualitatively dominant, convergent mixed methods design. Stage 1 involved codesigning an online platform with youth and parent coresearchers over a 4-month period and establishing a user registry of English-speaking youth (age 10-25 years) with T2D (diagnosed at age 18 years or younger) in Canada and their parents or guardians. In stage 2, up to 50 youth were purposively selected from the registry to complete baseline mental health measures, followed by 12 content modules using diary and arts-based response methods. Inductive thematic and mixed methods analysis will inform stage 3. Up to a third of the stage 3 participants will be purposively selected to identify priorities for mental health research and care and codesign arts-based KT resources to impart critical research findings to stakeholder groups identified with participants and youth coresearchers. This is an experimental modality for data collection, and participant numbers may be fewer; however, methodological insights regarding engagement will be collated and published to support digital strategy in future work.

**Results:**

We recruited 4 youth coresearchers to codesign the BrightSpark online platform—Canada’s first virtual living lab for youth and families with T2D—establishing an embedded registry of youth with T2D and creating the educational content of the 12 modules for the research. Recruitment and data collection began in March 2024 and concluded in September 2025. We anticipate study completion by January 2026.

**Conclusions:**

Youth-onset T2D presents a significant challenge to families and health systems, with less than 30% of youth with T2D achieving treatment targets. Self-management in youth with T2D is further complicated by psychosocial morbidity, identity formation, stigma, blame, shame, historical oppression, and structural barriers to health. This study will contribute a sustainable and novel structure to understand this issue, providing opportunities to generate critically needed understandings of youths’ mental health experiences to advance family-centered research and care.

**International Registered Report Identifier (IRRID):**

DERR1-10.2196/83865

## Introduction

### Background

Type 2 diabetes (T2D) is a complex chronic disease that affects an increasing number of youth and impacts the physical, emotional, and mental well-being of youth and families. Worldwide, the incidence of T2D in youth has risen sharply over the past two decades, particularly among marginalized and low-income populations, reflecting broader trends in obesity and health inequities [1-3]. In 2016, the American Diabetes Association highlighted an urgent need for patient-centered research and care in T2D, which presents as a more aggressive disease in youth [4,5]. Compared to adult-onset T2D, youth-onset T2D is characterized by faster beta cell decline; earlier insulin dependence; and higher risk of complications, such as nephropathy and retinopathy, within a few years of diagnosis [3,6].

The need for youth-centered research is amplified by knowledge that 30% of youth with T2D experience comorbid mental health challenges that are poorly understood and rarely prioritized in clinical practice guidelines, despite their negative impact on self-management [6-9]. These mental health challenges commonly include depression, anxiety, diabetes distress, and disordered eating behaviors, which can further complicate disease management and quality of life [10]. Despite increasing recognition of these challenges, few studies have explored how youth themselves conceptualize the relationship between diabetes and mental health [11]. Existing interventions are largely adult oriented or clinically driven rather than youth informed, limiting their effectiveness and relevance [6,12].

The importance of this gap has been reinforced by a participant advisory group (PAG) associated with our collaborating research team [5], which confirmed that mental health is a priority for youth with T2D and called for greater prioritization in research [13]. Yet, no qualitative or arts-based research exists in this area. This suggests an ongoing need for robust youth-centered research in T2D [6,7]. This proposal responds to this need through the cocreation of a living lab (ie, collaborative user-centered research environments that engage patients, clinicians, and researchers in codeveloping, testing, and refining real-world solutions to health care challenges) [14,15] for mental health in youth-onset T2D. We propose a study incorporating codesign, mixed methods [16-19], and arts-based knowledge translation (KT) [20-26] to open new avenues for patient-centered research in youth-onset T2D and mental health not previously considered.

### Youth-Onset T2D and Mental Health

The high incidence of youth-onset T2D in Manitoba (ie, >20/100,000/year) may serve as an early indicator of trends in other jurisdictions; the incidence of youth-onset T2D is highest in Manitoba but is growing across Canada [27]. In Manitoba, fewer than 30% of youth with T2D have achieved treatment targets of hemoglobin A1c (HbA1c)<7% despite the majority receiving specialized tertiary care—a comparable finding Canada-wide [27,28]. A critical factor influencing this finding is that we lack evidence on youth perspectives and experiences influencing their self-management and treatment outcomes. What is known is that self-management in youth with T2D is complicated by psychosocial morbidity, identity formation, stigma, blame, shame, historical oppression, and structural barriers to health facing minority groups disproportionately affected by T2D [29,30]. These factors increase the likelihood of short- and long-term complications [5,9,31,32], highlighting a need to better understand youth experiences, particularly in relation to mental health.

Mental health comorbidities, including psychiatric disorders and diabetes-specific psychological issues, affect 30% of youth with diabetes [33] but are often not prioritized by clinical care teams [34]. Compared to youth with type 1 diabetes (T1D), youth with T2D face heightened risk of early-onset depression [31,35], and the prevalence of substantial depressive symptoms in youth with T2D is 15%-22%—twice as high as youth with T1D [32]. These comorbidities are associated with higher risk of adverse microvascular and macrovascular complications [36]. Anxiety, fear [30,37], distress, hopelessness, and poor quality of life are prevalent [7,27] and impede self-management targets designed to reduce these comorbidities. Disconnects between youth and care provider priorities may exacerbate low rates of self-management and poor attendance in clinical care visits [35]. However, awareness of the complex relationship between mental health and T2D is lacking [38] and contributes to “diagnostic overshadowing” (ie, stigma in action) [39].

Clinical research into the mental health of youth with T2D is grounded in quantitative cross-sectional studies [40]; existing qualitative research into youth mental health in T2D is limited. Notably, Wicklow et al [41] investigated the determinants of renal complications in youth with T2D by developing a PAG comprising youth and caregivers. They identified themes such as blame and shame, stress and mental health, self-care and systemic issue (food security, support) that enriched their study framework and data collection tools and provided significant insights into the design and processes of the study [41]. Given that patient-centered resource and service design hinges on understanding patients’ perspectives [40] and that patient-driven strategies can empower patients and improve adherence and treatment outcomes [40,42], building upon these initial qualitative findings is critical to moving toward patient-centered models of research and care that connect with youth [39,43].

### Living Labs in Child Health

Living labs are “virtual, collaborative spaces where users create, prototype, and test concepts, innovations, products and systems” [44]. Living labs emerged in the early 2000s as promising collaborative platforms [45,46] and are widely adopted in civil engineering and aging research [14,47,48]. They are often used to support aging-in-place initiatives but have not been widely used in pediatric environments or as knowledge exchange platforms [15]. For instance, a systematic review of living labs for child health [15] provided evidence for living lab approaches for inclusive environments and to reduce health inequalities among vulnerable residents and regions [15]. However, initiatives drawing from living lab principles are poorly reflected in the extant literature.

Although various understandings of living labs have emerged in the literature, the core attributes of living labs have been identified in previous reviews. Technological infrastructure, stakeholder infrastructure, and community and end-user involvement have been identified as key characteristics of living labs [15]. Multimethod approaches, multiple users, user engagement, a real-life setting, and cocreation of an innovation environment are cited as paramount characteristics within the *Living Lab Methodology Handbook* [46]. Various user roles have also been identified, such as developer, contributor, and tester of an innovation; however, previous reviews of living labs demonstrate that although various roles for users can exist, there is an overreliance on the user as a tester of an innovation. Opportunities for users to be involved as developers or contributors to an innovation, or contribute to more bidirectional knowledge exchange, have been less acknowledged [15]. Considering the diversity, structural disadvantage, and regional variation in T2D across Canada, living labs are an ideal platform for patient engagement and integrated knowledge translation (iKT) in this area. They can support subgroup analysis and regionally specific codesign resources that respond to such variation.

Previous work piloted living labs in pediatrics and has shown their potential as a user-centered model for identifying priorities, collecting data, and codeveloping and evaluating resources and interventions [15] to ultimately support health care delivery and behavior modification [49-51]. Despite their relatively limited scope of application in relation to purpose and user roles, in our previous work, we began expanding this scope and using living lab principles as an innovative infrastructure to support patient registries for longitudinal qualitative and mixed methods research [14], enabling cultivation of rich insights into youth and family experiences in a manner not possible through traditional methods. Within the context of youth-onset T2D, registries and cohorts have been established to understand renal complications (eg, Improving Renal Complications in Adolescents with Type 2 Diabetes through Research [iCARE]) [5,9,27,52], and treatment options (eg, the Pediatric Diabetes Consortium) [53]. The current lab will serve as a complement through its focus on youth experiences and priorities.

### Study Purpose and Objectives

This study will help address the need for youth-centered research in youth-onset T2D and mental health. It will investigate youth perspectives and experiences influencing their self-management and treatment outcomes, as well as the complex relationship between mental health and T2D. Through longitudinal, qualitatively dominant mixed methods research, the study will generate understandings of youth experiences across a range of domains, helpful for driving new youth-centered inquiry and models of care. The study will also investigate the potential of living labs as an innovative platform for patient engagement and iKT. As such, the study will engage the following objectives to address these gaps:

- Objective 1: Create a virtual living lab platform with embedded registry of youth with T2D.
- Objective 2: Use this platform to generate critically needed understandings of youths’ mental health experiences.
- Objective 3: Identify youth priorities for research and care regarding mental health and T2D and communicate these priorities to providers and researchers in Canada using novel arts-based KT methods.

## Methods

### Study Design

This protocol was prepared in accordance with the GRAMMS (Good Reporting of Mixed Methods Studies) framework to ensure complete and accurate reporting [54] (see Multimedia Appendix 1 for the GRAMMS checklist). The research involves a three-stage longitudinal, qualitatively dominant, convergent mixed methods design that combines qualitative and quantitative data to explore the experiences of mental health and resiliency among youth with T2D using a virtual platform called BrightSpark as a living lab. Qualitatively dominant mixed methods research prioritizes the qualitative study strand, centering in-depth explorations that are supplemented by quantitative data. Stage 1 involved adapting a virtual platform for use as a living lab and recruiting youth with T2D and their parents or guardians to the participant registry through multi-phase purposive recruitment. Stage 2 involved recruiting up to 50 youth with T2D from the registry to participate in mixed methods data collection on their experiences of mental health and resiliency. Participants completed baseline and monthly content modules on topics identified as important to youth, including self-image and weight, substance use, wellness, coping, and resiliency. Stage 3 will involve using a convergent mixed methods design to analyze and integrate the qualitative and quantitative data collected in stage 2 to gain a comprehensive understanding of youth with T2D. Overall, this work aims to gain an in-depth understanding of the experiences of youth with T2D and provide support for their mental health and resiliency through exploratory, qualitatively dominant mixed methods research.

### Conceptual Framework

The Canadian Institute of Health Research (CIHR) Knowledge-to-Action Ethics framework [48] underpins this research and acknowledges often overlooked ethical considerations in the iterative processes of knowledge creation and translation. This is critical to this research, given the ethical nuances of research with youth, the situational oppression and stigma experienced by youth with T2D, and the underrepresentation of youth voices in T2D research. This framework provides a contextually grounded, inclusive, and respectful foundation for engaging and working with stakeholders. Its principles align closely with this research’s qualitatively dominant mixed methods design: for objective 1, it will guide the ethical development of a virtual living lab platform; for objective 2, it will ensure respectful and inclusive data collection on youth mental health experiences in the context of T2D; and for objective 3, it will inform the translation of youth priorities into research, practice, and policy through arts-based KT methods. Given the participatory, iterative, and translational nature of this research spanning digital innovation, patient engagement, and arts-based KT, the CIHR Knowledge-to-Action Ethics framework will ensure that ethical reflection, relational accountability, and action-oriented outcomes are integrated throughout each stage of the research.

### Study Setting and Inclusion Criteria

This study will be conducted among youth (10-35 years old) in Canada who received their T2D diagnosis before age 18 years. Data collection will be conducted mainly virtually on BrightSpark, an online living lab, which will allow the research team to reach potential participants across the country.

The inclusion criteria are as follows:

- Youth and young adults (10-35 years old) who received a T2D diagnosis as youths (on or before age 18 years)
- Those who speak English
- Those who live in Canada

### Recruitment Strategy

**Figure 1:**
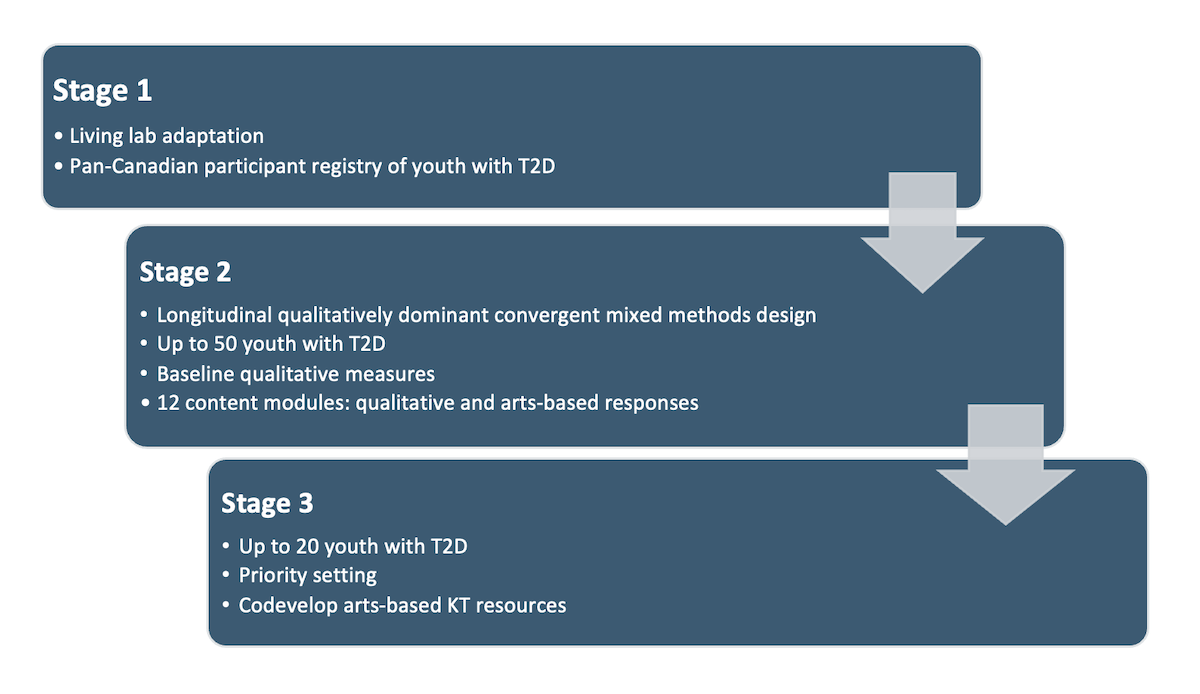
Research objectives outlined in three stages. T2D: type 2 diabetes.

The study is designed based on three research objectives, as outlined in Figure 1.

#### Objective 1: Create Canada’s First Living Lab Platform for Youth With T2D

##### Rationale and Foundational Work

Although patient registries are being developed across Canada by SPOR (ie, Strategy for Patient Oriented Research) networks to facilitate research engagement, patient-oriented research in youth-onset T2D is lacking.*:* Over 12 months, we engaged a PAG of youth, parents, and knowledge users (KUs) to establish platform utility. The PAG indicated that this platform is needed to connect, share experiences of mental health, and build T2D awareness. Youth desire an engaging social media style platform with gaming features, such as avatars and points, to incentivize engagement [55,56]. A systematic review [56] identified seven gamification strategies (goal setting, challenges, feedback, reinforcement, playfulness, progress comparison, social connectivity) that create persuasive system architecture to impact user behavior and experience, providing a strong rationale for their use in the lab [56,57]. A foundational systematic review [15] of living labs in child health was conducted, and the first codesigned clinical living lab for collaborative research and iKT in pediatric rehabilitation [14] enabled us to identify multimethod, user-centered strategies to further optimize platform design.

##### Codesigning an Engaging Platform

The technology company Recollective helped create the living lab by adapting an existing platform for cultivating online communities. Four youth coresearchers helped codesign the platform. The platform houses a registry where youth with T2D and their guardians (for consent) register in a panel before receiving research study invitations. After consent, registrants become activated participants who at study completion will return to the registry until contacted for a future study. Archive registration preserves all registry and study information between studies, protected by tiers of platform security and data encryption. The BrightSpark URL functions on any smart device (eg, tablet, phone) and presents as a social media app (eg, Facebook) to maximize usability. An evidence-based, youth-directed gamification strategy integrating the seven persuasive strategies [56,57] will incentivize use through points and other customized feedback approaches. Incentives will be redeemed within (eg, activate platform features) and beyond (eg, reimbursement) the platform to optimize engagement. System parameters will prevent incentive overuse for each time period.

##### Participants and Recruitment

English-speaking youth (10-35 years old) with T2D (diagnosed at age 18 years or younger) in Canada and their guardians (for consent) were invited to join the lab. A broad age range was selected to promote feasibility and enable meaningful subgroup comparisons. Our PAG and the literature reporting sociodemographic characteristics suggest little exclusion bias with English language restriction [5,27].

A research coordinator and graduate research assistant facilitated multiphase purposive recruitment through the Diabetes Education Resource for Children and Adolescents (DER-CA) in Winnipeg, Manitoba, and the national iCARE cohort. DER-CA staff shared study information via onsite posters and clinic visits. The research personnel provided study information to previously consenting iCARE participants using emails and phone numbers provided during iCARE recruitment*.* Study information was also shared through Facebook and Twitter advertising; Northern communities Buy/Sell; and relevant clinic sites in Canada. Snowball sampling, where participants, KUs, and coresearchers contact potential participants, is being used. Although potentially a source of bias, snowball sampling is appropriate here since T2D often occurs in high rates in communities, clustered in cohorts; the method is sensitive to power relations and social networks [58]. Prospective participants received the BrightSpark URL and created registry profiles with avatars or nonidentifying images. Registry data includes age, sex, gender, ethnicity, geographic location, and years since diabetes diagnosis.

##### Data Collection in the Living Lab

The platform has embedded data collection and codesign strategies for each study stage, including image-based response; real-time video and time stamping; audio recording; questionnaires; short, long, and diary-answer response formats; and forums.

#### Objective 2: Investigate Youth Experiences of Mental Health and Resiliency Through Longitudinal, Qualitatively Dominant Mixed Methods Research Within the Living Lab

##### Rationale

Longitudinal research using interpretive description and arts-based methods is important for generating robust understandings of dynamic youth experiences not captured through short-term studies; for informing iKT, given its emphasis on clinical application of data; and because arts-based methods are developmentally appropriate and efficacious and have been used successfully in a local sample of youth with T2D [22,59].

##### Participants, Sampling, and Recruitment

Up to 50 youth with T2D were purposively recruited from the BrightSpark registry on the basis of sex, gender, age, years since diagnosis, ethnicity, and geographic location since (1) there is a higher T2D incidence in young females than in males [39]; (2) age and gender impact mental health comorbidities and experiences [60]; (3) years since diagnosis impact the sense of mastery, which is associated with positive mental health [39]; and (4) there are disproportionate rates of T2D and compounding factors impacting mental wellness in minority and remote groups [27,29,39]. Purposive recruitment enables the study of intersectionality since socioeconomic and demographic categories are interacting, not distinct [61]. With the qualitative emphasis, no sample size calculation was performed; efforts are guided by data saturation [62-64] to enable credible subgroup comparisons.

##### Data Collection

In the first 2 months, baseline data, including sociodemographic and health data (see Table 1), were collected. These measures were selected based on their prevalence and PAG prioritization, validity, and reliability. The 17-question Diabetes Distress Scale assesses T2D’s emotional burden, self-management distress, and social and patient-provider relationship stress and has high reliability (α=.92) and discriminate validity [65]. The Centre for Epidemiological Studies Depression Scale is a 20-item, 4-point scale covering major dimensions of depression, with demonstrated validity from age 6 years through adulthood [66]. The Beck Anxiety Inventory is a 21-item, 4-point scale with strong reliability (α=.92) [67]. Over 12 months, participants also completed monthly content modules (ie, data collection modules; Table 2) including one narrative (video/audio/written) and one arts-based (eg, photographs, drawings) response to exploratory research questions on topics identified from research and PAG/coresearcher engagement. These qualitative data were collected as asynchronous qualitative freeform responses within the BrightSpark platform in response to the domain topic and introductory question. Integration will occur at the moderate level [16,68]: Quantitative data will contextualize qualitative and arts-based findings, inform subgroups for thematic analysis; the extent of missing data will inform the feasibility of future studies; and qualitative analysis will generate hypotheses for future studies within the lead investigator’s mixed methods research program.

**Table 1:** Descriptive characteristics and experiential domains.

Outcome	Data type	Measure/tool	Description
Participant sociodemographic characteristics	Quantitative	BrightSpark registry	Sex, gender, age, years since diagnosis, ethnicity, geographic location, family composition (eg, number of siblings, parents/guardian relationship, parents/guardian participant lives with)
Anthropometric measures	Quantitative	Height and weight (self-reported or measured)	Assesses body composition or general physical status
Glycemic management	Quantitative	Most recent A1c test (self-reported or medical record)	Last-known A1c value (%) to assess blood glucose control over the past 2-3 months
Treatment	Quantitative	Self-reported	Lifestyle interventions (eg, diet, exercise/activities) and medications
Mental health comorbidity	Quantitative	Diabetes Distress Scale [65], Centre for Epidemiological Studies Depression Scale [66], Beck Anxiety Inventory [67]	Assesses diabetes-related distress levels, risk of depression, and anxiety levels
Experiences and challenges	Qualitative	Questionnaire, diary-answer responses, real-time videos, audio recordings, image-based responses	Inductive thematic analysis identifying participant experiences
Coping strategies/facilitators	Qualitative	Questionnaire, diary-answer responses, real-time videos, audio recordings, image-based responses	Themes identified through interpretive description [69] describing strategies/supports used by participants
Integrated insights	Mixed	Joint displays and narrative integration	Synthesizes and juxtaposes quantitative and qualitative findings for a holistic interpretation

**Table 2:** Monthly content modules for qualitative data collection.

Module	Content
1	Receiving a T2D^a^ diagnosis
2	Managing T2D, anxiety, and stress
3	T2D medication by needle or mouth and low and high blood sugar
4	Relationships with family
5	Relationships with friends (social relationships)
6	Relationships with health care professionals
7	School, sports, and work
8	Self-image and weight
9	Substance use
10	Wellness, coping, and resilience
11	Food security and choices
12	Balancing everyday nourishment with special occasions

^a^T2D: type 2 diabetes.

##### Procedures

A visual timeline prompted activated participants to complete baseline measures, which unlocked the qualitative content modules and a list of free mental health support resources. Participants could then select a minimum of one and a maximum of three modules monthly for 12 months in any order, with the option to suggest new topics to identify additional priorities. Participants provided qualitative responses (written or audio) in response to the module-specific research questions; provided an accompanying artistic response, if desired (eg, photograph, drawing); and received additional evidence-based information following their response provision within the platform. Participants received CA $20 (approximately US $14) honorariums for each module completed.

##### Data Analysis

A trained graduate research assistant and research coordinator will use a con*v*ergent mixed methods data analysis approach. Quantitative data will be descriptively analyzed using IBM SPSS to summarize participant sociodemographic characteristics and outcome data, as described in Table 1. Qualitative data will be inductively analyzed using interpretive description [69] in MAXQDA mixed methods software to identify themes reflecting participant experiences and insights. System-generated transcripts will be read repeatedly to gain a sense of the whole, prior to coding line by line. Open coding will ensue, commencing identification of meaning units at the level of word clusters or sentences, and associated definitions for codes will be created in a codebook. Codes from the first three transcripts will inform a coding framework to apply to the remaining transcripts. New data from the remaining transcripts will refine the coding framework [69]. Codes will be examined for frequency, with focus on within- and across-case coverage, and tentatively grouped into themes. Themes will be substantiated with codes and quotes and reviewed before labeling to enable flexibility with conceptualization and to ensure subgroup comparisons (eg, gender comparisons) are reflected. Cutoff categories for baseline measures will be examined to inform subgroups (eg, low, moderate, high distress); themes will be clustered and thematic labels modified, as needed [70]. Arts-based data will be analyzed by key content, constituent elements, and tone using a framework successfully applied in our previous work [71] and handled holistically with accompanying narrative data. Participants will validate their interpretation. The module selection order will be tallied and descriptively analyzed to suggest content prioritization. Quantitative and qualitative findings will be integrated in joint displays, enabling meta-inferences to be drawn [72-74]. Analysis will generate hypotheses, while producing rich and expressive representations of mental health and T2D to inform stage 3. Recognized trustworthiness [75] and mixed methods legitimation [76] criteria will assure quality. Strategies will include member checking (credibility), analytic memos (dependability), substantive linking of interpretations with data (confirmability, meta-inferential), and considering alternative explanations (authenticity).

#### Objective 3: Identify and Communicate Youth Priorities Using Arts-Based KT

##### Rationale

A 2016 consensus document [7] for youth-onset T2D emphasizes the need to prioritize outcomes other than glycemic control. Our PAG identified mental health as a key research domain, yet youth priorities for mental health research and care in T2D remain unknown. As such, we aim to identify research and care priorities of youth with T2D regarding mental health and resiliency. Our research has shown the power of iKT in identifying priorities and communicating findings through arts-based KT (eg, communicating research using storylines based on family experiences) [20,24,26,77]. Such KT resources in youth-onset T2D are needed but lacking.

##### Participants and Sampling

Up to a third of the participants from stage 2 will be purposively sampled from the registry by age, gender, geography, mental health status, and engagement (eg, >90% module completion in stage 2) to provide sufficient diversity to generate credible findings regarding priorities and to inform KT resource development. This aligns with the Delphi technique’s emphasis on participant qualities over representative samples [78].

##### Procedures

We will use card sorting [79] with a modified Delphi technique [78]—common and robust priority-setting method—to identify youth priorities from stage 2. Youth will be recruited to a forum within BrightSpark and provided with a series of cards, each representing a theme from stage 2 (first Delphi round). Participants will (1) rank-order the cards by priority (second Delphi round), (2) provide written/verbal feedback and identify priorities not represented (member checking), and (3) amend their ranking following feedback (third Delphi round) [78]. Rank orders will be tabulated to determine up to 10 priority themes. Participants will be led through the following seven steps to codesign up to four arts-based KT resources and two infographics:

- Step 1: Gain KT familiarity, including KT goals relevant to the research (eg, raise awareness, knowledge) [80,81].
- Step 2: Identify target audiences based on the KT principle of tailoring to stakeholder groups (eg, health provider) and evidence of effectiveness of strategies for each group [82].
- Step 3: Identify key messages based on the KT principle of aligning with target audiences and strategies [81-83].
- Step 4: Determine delivery modality (eg, video, comic) in reference to the Archibald arts-based KT classification framework [77] and evidence of effectiveness of arts-based KT strategies [23,84].
- Step 5: Provide storyboard content for iterative revision [24].
- Step 6: Provide feedback at key development stages.
- Step 7: Identify dissemination channels based on preferences and evidence of online analytics [85,86].

At this stage, participants will be provided CA $100 (approximately US $72) honorariums for participation in this stage.

##### Data Collection and Analysis

We will use three-stage card sorting in the modified Delphi technique for prioritization, video/audio time stamping with narrative feedback for KT development, forum question-and-feedback sessions anchored to each step of the development process, and an inverted rank-order analysis used in our previous work to consolidate priorities [87]. A research assistant will conduct directed qualitative content analysis [88] of forum data using MAXQDA, with purposive subgroups as analytic units focusing on manifest content. A formative coding matrix will be applied to each forum thread, corresponding to each step of KT development. Interim analysis will direct forum questioning to inform the KT resources.

##### Knowledge Translation Plan

We engaged stakeholders from the onset of study conceptualization [40,42], leveraging their strengths and perspectives, while responding to families’ prioritization of mental-health and a critical knowledge gap. We will innovate on iKT by using evidence-based KT principles to codevelop arts-based KT resources (stage 3) to mobilize findings. Dissemination through the lead author’s website and YouTube channel will be informed by YouTube and Google Analytics [81]. Resources will be submitted to the CIHR Institute of Human Development, Child and Youth Health (IHDCYH) Talks competition (video form) and resource centers. BrightSpark aligns with the patient-oriented research movement in Canada, providing a rare opportunity to consolidate and communicate youth priorities in T2D to springboard future iKT and youth-centered research of collaborators. It will provide the infrastructure to scale up other investigations into youth-onset T2D and methodological guidance for other living lab initiatives nationally and internationally, with applicability to other chronic disease contexts.

### Ethical Considerations

This study was reviewed and approved by the University of Manitoba Health Research Ethics Board (approval number HE2022-0396) and the Shared Health Approval Committee for Privacy, Impact and Access in Research (approval number SH2023:042). Written consent of participation, to record in stage 3 and to use and disseminate data, was collected from all participants on the virtual living lab platform prior to participation in stages 2 and 3. All identifying information will be removed from the data collected through narratives and art-based modules in stage 2 of the project. Similarly, recordings of the group discussion in stage 3 will be destroyed after transcription, and transcriptions will be kept for 5 years in a secure locked file on a secure and password-protected device only accessible to the principal investigator (author MA) and identified personnel of the research team. The module content and presentation were thoughtfully designed to reflect a supportive orientation and were informed with participant advisory input; context-relevant mental health resource lists were made available to participants at multiple time points within the platform, associated with each module.

## Results

A name and logo for the living lab was crowdsourced on Hatchwise, and the research team voted to select the name BrightSpark, Care Lab and the logo. Four youth coresearchers and the research team codesigned BrightSpark for youth and families with T2D and created the educational contents of the 12 modules for the research. Data collection and recruitment began in March 2024 and concluded on September 23, 2025, with analysis ongoing through January 2026. Findings from this study will be disseminated through peer-reviewed publications, presentations at national and international conferences, arts-based KT, and digital and social media platforms targeted at youth and health care providers.

## Discussion

### Summary

This study anticipates that youth with T2D will report a high prevalence of mental health challenges, including stress and anxiety, which are likely to influence their self-management behaviors and treatment outcomes [29,30]. The ordered identification of pertinent response modules, as indicated by participant-led selection and completion, will provide insight into possible domains of perceived relevance for youth, guiding future exploratory and intervention research. It is also expected that the study will provide insights into the feasibility and acceptability of a virtual youth-centered platform for research participation, highlighting potential strategies for engaging this population in patient-centered care and research.

The existing literature underscores that mental health challenges in youth with T2D are underrecognized and poorly addressed, yet they have substantial implications for adherence, glycemic control, long-term complications, and overall wellness [7-9,29,30,89]. Although quantitative research is generating evidence around the prevalence of such challenges, qualitative investigations are less common. A recent interpretive descriptive study [13] with 22 participants diagnosed with T2D before 18 years of age emphasized the pervasive impacts of mental health on T2D experiences and management, highlighting the intersections between blood sugar stability, mood, mental health, and considerations related to growth and development. Similarly, a focus group study [89] involving eight First Nation adolescents identified common experiences around stigma and shame, and the associated weight of managing a T2D diagnosis. However, the specific domains and facets of such mental health experiences have not been explored. This study will significantly extend the limited qualitative investigations emerging in this area.

The anticipated findings from this study may extend prior work by illuminating the nuanced ways in which mental health intersects with self-management in youth, as well as by demonstrating the potential of technology-mediated approaches for capturing these experiences. Living labs have shown promise in adolescent populations for both research participation and health interventions [90], but evidence on their application for qualitatively dominant research with youth with T2D is limited. This work anticipates generating understandings and perspectives that will inform patient-centered strategies that connect with youth. Concurrently, the work will provide insight into the utility of such a virtual platform for youth-centered research, which has high value in an increasingly user-centered and technologically proficient society.

### Strengths and Limitations

A major strength of this study is the use of a qualitatively dominant, convergent mixed methods approach, which allows for the integration of quantitative and qualitative data to provide a more comprehensive understanding of mental health experiences and self-management behaviors in youth with T2D. This approach can reveal patterns not apparent through a single method and support comprehensive understandings, enhancing the validity of the findings. Additional strengths include the use of a virtual platform, which may increase accessibility for diverse participants, and the direct engagement of youth and the PAG ensures that the findings accurately reflect and are relevant to their lived experiences.

Limitations include a potential for selection bias, as youth with higher digital literacy or motivation may be more likely to participate. However, given that the virtual living lab was codesigned with youth and the PAG representing diverse experiences with technology, socioeconomic backgrounds, and health literacy levels, selection bias was likely reduced. Through iterative codesign sessions, the PAG informed the platform’s accessibility features, plain-language guidance, navigation, and tone ensuring inclusivity and ease of use. The embedded codesign principles and continuous feedback loops lowered potential digital participation barriers and promoted equitable engagement among youth and parents who might otherwise be underrepresented due to varying comfort levels with virtual platforms or differing levels of intrinsic motivation.

Codesigning the virtual living lab environment with youth and the PAG will also serve to mitigate potential biases related to self-reported data and interpretation of mixed methods data integration. The involvement of advisors in shaping the data collection tools, question framing, and engagement formats will foster psychological safety and participant ownership, which are conditions that reduce social desirability bias and encourage authentic sharing of experiences [91]. The inclusion of multimodal reflection options (eg, written, audio, or video responses) will allow participants to provide input in real time or asynchronously, lessening reliance on retrospective recall and associated recall bias. Furthermore, our engagement with youth and the PAG in defining the key constructs and indicators will ensure conceptual alignment between qualitative and quantitative components, thereby strengthening the methodological coherence.

### Future Directions

The insights gained from this study are expected to inform the development of targeted, patient-centered interventions for youth with T2D that address both mental health and self-management needs. Future research could explore additional longitudinal outcomes, evaluate intervention effectiveness, and examine strategies to sustain engagement in digital platforms. Additionally, findings may inform clinical guidelines and support the integration of mental health assessment and support into routine T2D care for youth and adolescents. Engaging participants in the dissemination process, such as through cocreated summaries, arts-based KT resources, or infographics, may further enhance the relevance and impact of the findings.

### Conclusion

By exploring the mental health experiences of youth with T2D and the feasibility of a virtual living lab for research engagement, this study aims to generate actionable insights to inform youth-centered care and research strategies. Through this innovative living lab, this study will contribute a sustainable structure to understanding and supporting youths’ mental health needs, advancing both research and care practices.

## Appendix

Multimedia Appendix 1 GRAMMS (Good Reporting of Mixed Methods Studies) checklist.

Multimedia Appendix 2 Peer review report from SickKids Canada New Investigators Research Grants Committee.

## Abbreviations

CIHR: Canadian Institute of Health Research
DER-CA: Diabetes Education Resource for Children and Adolescents
GRAMMS: Good Reporting of Mixed Methods Studies
iCARE: Improving Renal Complications in Adolescents with Type 2 Diabetes through Research
IHDCYH: Institute of Human Development, Child and Youth Health
iKT: integrated knowledge translation
KT: knowledge translation.
KU: knowledge user
PAG: participant advisory group
T1D: type 1 diabetes
T2D: type 2 diabetes

## Data Availability

The datasets generated during this study are not available.
