# Supplementary material for: A Virtual Living Lab Platform Codeveloped for Mental Health in Youth-Onset Type 2 Diabetes (BrightSpark Care Lab): Protocol for a Mixed Methods Study
Source: JMIR Res Protoc. 2026 Jan 19;15:e83865. doi: 10.2196/83865 (PMC12865352; doi:10.2196/83865)
Supplement: Multimedia Appendix 1 [file resprot_v15i1e83865_app1.pdf]

GRAMMS (Good Reporting of Mixed Methods Studies) - O'Cathain A, Murphy E, Nicholl J. The quality of mixed methods studies in health services research. J Health Serv Res Policy. 2008;13(2):92-98

Reporting Item	Where in Manuscript
(1) Describe the justification for using a mixed methods approach to the research question	Methods: quantitative data will contextualize qualitative and arts-based findings, inform subgroups for thematic analysis; extent of missing data will inform feasibility of future studies; and qualitative analysis will generate hypotheses for future studies within the lead investigator's mixed methods research program.
(2) Describe the design in terms of the purpose, priority and sequence of methods	Methods: The research project involves a 3-stage longitudinal, qualitatively driven convergent mixed methods design that combines qualitative and quantitative data to explore the experiences of mental health and resiliency among youth with type 2 diabetes (T2D) using a virtual platform called BrightSpark, as a living lab. Qualitatively driven mixed methods research prioritizes the qualitative study strand, centering in-depth explorations which are supplemented by quantitative data.
(3) Describe each method in terms of sampling, data collection and analysis	Methods:<br><br>Objective 1<br>*Recruitment:* **...** Snowball sampling, where participants, KUs and co-researchers contact potential participants, is being used.<br><br>*Data Collection in the Living Lab:* The platform has embedded data collection and co-design strategies for each study stage, including image-based response, real-time video and time stamping, audio recording, questionnaires; short, long, and diary-answer response formats, and forums.<br><br>Objective 2<br>*Participants, Sampling & Recruitment:* Up to 50 youth with T2D were purposively recruited from the BrightSpark registry on the basis of sex, gender, age, years since diagnosis, ethnicity, and geographic location

	since: there is higher T2D incidence in young females than males [39]; age and gender impact mental health co-morbidities and experiences [60] years since diagnosis impacts sense of mastery, which is associated with positive mental health [39]; and there are disproportionate rates of T2D and compounding factors impacting mental wellness in minority and remote groups [27,29,39]. Purposive recruitment enables study of intersectionality since socioeconomic and demographic categories are interacting, not distinct [61]. With the qualitative emphasis, no sample size calculation was performed; efforts are guided by data saturation [62–64] to enable credible subgroup comparisons.<br><br>*Data Collection:* In the first 2 months, baseline data including socio-demographic and health data (see Table 1) was collected. These measures were selected based on their prevalence and PAG prioritization, validity, and reliability. The 17-question Diabetes Distress Scale assesses T2D's emotional burden, self-management distress, social and patient-provider relationship stress and has high reliability (a=0.92) and discriminate validity [65]. The Centre for Epidemiological Studies Depression Scale is a 20-item, 4-point scale covering major dimensions of depression with demonstrated validity from age 6 years through adulthood [66]. The Beck Anxiety Inventory is a 21-item, 4-point scale with strong reliability (a=0.92) [67]. Over 12 months, participants also completed monthly content modules (i.e., data collection modules, Table 2) including 1 narrative (video/audio/written) and 1 arts-based (e.g., photographs, drawings) response to exploratory research questions on topics identified from research and PAG/co-researcher engagement.<br><br>*Data Analysis:* A trained graduate research assistant and research coordinator will employ

	a convergent mixed methods data analysis approach. Quantitative data will be descriptively analyzed using SPSS to summarize participant socio-demographic characteristics and outcome data as described in Table 1. Qualitative data will be inductively analyzed using interpretive description [69] within MAXQDA mixed methods software to identify themes reflecting participant's experiences and insights.<br><br>Objective 3<br>*Participants and Sampling:* Up to a third of participants from stage 2 will be purposively sampled from the registry by age, gender, geography, mental health status and engagement (e.g., $\geq$90% module completion in stage 2), to provide sufficient diversity to generate credible findings regarding priorities and to inform KT resource development.<br><br>*Data Collection and Analysis:* We will use 3-stage card-sorting in the modified Delphi technique for prioritization, video/audio timestamp with narrative feedback for KT development, forum question-and-feedback anchored to each step of the development process, and an inverted rank order analysis used in our previous work to consolidate priorities [88]. In MAXQDA, a research assistant will conduct directed qualitative content analysis [89] of forum data with purposive subgroups as analytic units focusing on manifest content. A formative coding matrix will be applied to each forum thread, corresponding to each step of KT development. Interim analysis will direct forum questioning to inform the KT resources.
(4) Describe where integration has occurred, how it has occurred and who has participated in it	Methods: Integration will occur at the moderate level: [16,68] quantitative data will contextualize qualitative and arts-based findings, inform subgroups for thematic analysis; extent of missing data will inform

	feasibility of future studies; and qualitative analysis will generate hypotheses for future studies within the lead investigator's mixed methods research program.<br>...<br>Quantitative and qualitative findings will be integrated in joint displays, enabling meta-inferences to be drawn
(5) Describe any limitation of one method associated with the present of the other method	Methods:<br><br>Objective 1<br>*Recruitment:* ... While potentially a source of bias, snowball sampling is appropriate here since T2D often occurs in high rates in communities, clustered in cohorts; the method is sensitive to power relations and social networks [58].<br><br>Strengths and Limitations: Limitations include a potential for selection bias, as youth with higher digital literacy or motivation may be more likely to participate. ... Co-designing the virtual living lab environment with youth and the PAG also serve to mitigate potential biases related to self-reported data and interpretation of mixed methods data integration. The involvement of advisors in shaping the data collection tools, question framing, and engagement formats fosters psychological safety and participant ownership, which are conditions that reduce social desirability bias and encourage authentic sharing of experiences [92]. The inclusion of multimodal reflection options (e.g., written, audio, or video responses) allow participants to provide input in real time or asynchronously, lessening reliance on retrospective recall and associated recall bias.
(6) Describe any insights gained from mixing or integrating methods	Future Directions: The insights gained from this study are expected to inform the development of targeted, patient-centered interventions for youth with T2D that address both mental health and self-management needs. Future research could explore additional longitudinal outcomes, evaluate

	intervention effectiveness, and examine strategies to sustain engagement in digital platforms. Additionally, findings may inform clinical guidelines and support the integration of mental health assessment and support into routine T2D care for youth and adolescents.
