# Supplementary material for: A Virtual Living Lab Platform Codeveloped for Mental Health in Youth-Onset Type 2 Diabetes (BrightSpark Care Lab): Protocol for a Mixed Methods Study
Source: JMIR Res Protoc. 2026 Jan 19;15:e83865. doi: 10.2196/83865 (PMC12865352; doi:10.2196/83865)
Supplement: Multimedia Appendix 2 [file resprot_v15i1e83865_app2.pdf]

# Review for
# Mandy Archibald

Application ID (NI22-1046R)

SickKids

Review Summary

**NI22-1046R**

**Reviewer 1**

## Reviewer Comments

**Research Impact Comments:**

This proposal to establish Canada's 1st virtual living lab to monitor and help ameliorate adverse mental health in Canadian young type 2 diabetics has great impact to a large population of T2D youth. The rationale for this study is very strong given (i) the rising incidence of T2D in Canada, (ii) T2D specifically (vs T1D) is associated with depression in 15-22% of T2D patients, and (iii) 30% of T2D youth experience co-morbidities with respect to mental health challenges which was top priority with participant advisory group. Moreover, only 30% of T2D youth are achieving their end targerts of HbA1c < 7%. So strong rationale from a mental and endocrine standpoint. The methods in this study are established by the PI, and the potential to improve the youth's quality of life through this living lab and modules is very high.

**Scientific Methodology Comments:**

This resubmitted SickiKids grant addresses many of the outstanding concerns from previous reviewers. It provides a validated 3-stage longitudinal convergent mixed methods research study: stage 1 - HIPPA compliant virtual living lab involved creating a large registry of youth with T2D. In stage 2, 50 T2D youths are recruited for mental health measures and intervention content modules, while stage 3 involved recruiting 20 youths to address priorities for mental health research and care. Previous issues with feasibility and mitigation strategies with respect to recruitment have now been adequately addressed. The feasibility of the study is enhanced with commercial partnerships (ie. Recollective Technology) and a great clinical research team drawn from DER-CA clinicians and PIs from iCARE. The platform is engaging, the data collection is real-time, and the target participation group (ages 10-18 years) is broad and impactful. More details/criteria is needed to asses how the arts-based data will be assessed/quantified for efficacy? Also, what is the exclusion criteria for objectives 1-3? While data will be assessed based on a number of identified covariates, geographical location (ie. postal codes) was not included. This would be informative not only for assessment of socioeconomic status, but also insightful in comparing rural vs city and proximity to fast food, community centers, etc. It also remained unclear how specifically participants would be selected for Objectives 2 and 3 - especially Objective 3.

**Innovation/Originality Comments:**

As previously mentioned, this is a highly innovative and original proposal creating the first virtual living lab for T2D youth. The PI has a track record in efficacious registry formation for Renal disorders and from Pediatric populations. This platform is supported by several research teams and created based on the priorities outlined by iCARE participant advisory group (PAG).

**Knowledge Translation Comments:**

The KT plan proposed was well described. It is apparent that the PI has connected with the correct networks of physicians and patients, including Canadian pediatric and endocrine groups. The PI plans to dissemminate their research outcomes at Canadian pediatric conferences, CIHR-IHDCYH talks competetion, and on validate YouTube channels. It is also very impressive that the PI has engaged First Nations Resource Center for recruitment and dissemination.

**Mentorship, Research, and Environment:**

The PI is surrounded by a great team with the College of Nursing, CHRIM, and DREAM at U Manitoba. The PI provides Table 3 as evidence-based mentorship plan (Table 3) which is a true asset to this proposal. A strong letter of support from Dr. Dyck (Dean of Nursing, U Manitoba) describes full institutional support. Furthermore, Dr. Archibald has great mentorship support as described in two strong letters from Dr. Woodgate (CRC Tier 1, expertise in KT and mental health), and Dr. McGavock (DREAM Co-PI, expertise in T2D). An impressive letter of support was also provided by Jackie Moore, an Objibwee Co-Chair of the iCARE PAG. Finally Dr. Sellers (pediatric endocrinologist) is also listed as a collaborator, expert, and mentor to the PI which is very supportive.

**NI22-1046R**

**Reviewer 2**

# Reviewer Comments

**Research Impact Comments:**

This proposal is addressing a topic of increasing importance namely mental health problems in youth with type 2 diabetes, a group which is increasing in number. Addressing mental health needs is crucial to improve self-management and overall outcomes in this population. Many studies have described higher prevalence of mental health comorbidities in type 2 diabetes, but patients were most often adults and very few (if none) have examined pediatric patient perspective and priorities regarding mental health research.

The research team has widened their recruitment to include patients outside of Manitoba thus increasing generalizability.

Given the iKT approach, this study will generate deliverables (art-based resources) that can be readily used to raise awareness regarding mental health problem, which is a first key step to identify vulnerable individuals and provide support. It will also provide valuable information on research priorities which will benefit the scientific community and a comprehensive representation of mental health in youth with diabetes, which should be of value for clinicians. In addition, the virtual living-lab platform with embedded registry offers incredible opportunities for future research endeavours in this population.

Overall, the impact of this research will be strong.

**Scientific Methodology Comments:**

The weaknesses identified in previous application have been addressed. Details on recruitment to the registry are provided. Patients will be approached mainly through the iCARE cohort, n=322, with the 2.0 cohort funded for five years thus increasing feasibility and DER-CA, a specialty clinic with over 300 patients. The justification of the snowball sampling method is well argued (clustering of eligible participants in communities).
The rationale for the virtual living lab model is better explained and supported by work conducted with the patient advisory group and a systematic review by PI Archibald. The platform is now well described with justification of the gamification approach. The co-design of the platform with youth co-researchers is definitely a strength in this proposal.
The use of mixed methods to investigate youth experiences of mental health and resiliency is appropriate in this context allowing to quantify level of mental health problems using standardized widely used questionnaires (Diabetes Distress Scale, Centre for Epi Depression Scale, Beck Anxiety Inventory) in relation to qualitative and art-based data. The approach is, once again, well described and supported by the literature. Step 3, which consists in the co-development of art-based KT to communicate priorities using a modified Delphi approach, is equally well detailed.
In this type of study, the risk is to voice the opinion of the loudest individuals, which may not represent more marginalized people. However, using purposeful sampling will address this issue and increase generalizability of findings.
Very few weaknesses in my opinion.

**Innovation/Originality Comments:**

The whole concept is novel in itself and can open the field to new research approaches.
Building a virtual living-lab platform is truly inspiring and probably something to develop in the future for other young populations who were born in the era of technology.

**Knowledge Translation Comments:**

The project integrates knowledge translation into its overall approach. Engagement of various stakeholders including clinicians, researchers and most importantly youth and families is very strong in this proposal.
Deliverables are clear with ‘traditional’ scientific publications/presentations as well as art-based KT material/infographics that can be disseminated to a wide audience through different channels (team very well connected). Given the importance of mental health in youth with diabetes and lack of resources, this work could also reach policymakers to sensitize them to this problem.

**Mentorship, Research, and Environment:**

There is absolutely no weakness that I could see.
Very strong PI with a background and publication track record that show that she has the knowledge and expertise to conduct such research.
She is supported by a multidisciplinary team, including patient partners and a knowledge keeper from the Ojibwe community, that covers all aspects of the proposal.
There is a strong support letter from her institution (80% protected research time, 150,000$ startup funds) and one of the best mentorship plan I have seen.

SickKids



**NI22-1046R**

**Reviewer 3**

## Reviewer Comments

**Research Impact Comments:** Given the lack of research into the mental health experiences of youth with T2D, and the potential impact of the quality of life of a large portion of Canadian Children, this is a novel and interesting project. The inclusion of youth throughout as experts, is a strength of this proposal and will ensure its outcomes are in line with what patients and other stakeholders have been demanding. The use of longitudinal qualitative work is innovative, and likely the best design for the topic at hand. The concept of a living lab is interesting and novel, with interesting lessons learned for other pediatric and mental health researchers in Canada.

**Scientific Methodology Comments:** This is a very throughout, thought out and justified study. The use of innovative and patient-centred methods is highlighted throughout, strengthening the proposal. The use of several illness specific instruments (Beck, CES-D, etc) without strength-based measures or functioning measures may be an area to explore further, particularly given the strong focus on patient co-design.

**Innovation/Originality Comments:** Interesting and well-designed project. Clearly building on, and leveraging, the applicants program of research and expertise.

**Knowledge Translation Comments:** Excellent overview of KT, with links to specific work the applicant has already led. Strong integration of youth with lived experience, including as co-I's.

**Mentorship, Research, and Environment:** Clearly a world-class training environment for the applicant. The mentor is a leader in the field internationally. The applicant has been highly successful in receiving funding, has an exemplary publication/presentation record, and has an exemplary track record with graduate students.
There is high likelihood of success for the project and to support the applicant in becoming an independent scientist.
The mentorship plan is extremely detailed, and well supported by the letters from mentors.
